# Active 3D positioning and imaging modulated by single fringe projection with compact metasurface device

**DOI:** 10.1515/nanoph-2023-0112

**Published:** 2023-04-04

**Authors:** Xiaoli Jing, Yao Li, Junjie Li, Yongtian Wang, Lingling Huang

**Affiliations:** Beijing Engineering Research Center of Mixed Reality and Advanced Display, School of Optics and Photonics, Beijing Institute of Technology, Beijing 10081, China; Beijing National Laboratory for Condensed Matter Physics, Institute of Physics, Chinese Academy of Sciences, Beijing 100191, China

**Keywords:** depth sensing, geometric phase, metasurface

## Abstract

Three-dimensional (3D) information is vital for providing detailed features of the physical world, which is used in numerous applications such as industrial inspection, automatic navigation and identity authentication. However, the implementations of 3D imagers always rely on bulky optics. Metasurfaces, as the next-generation optics, shows flexible modulation abilities and excellent performance combined with computer vision algorithm. Here, we demonstrate an active 3D positioning and imaging method with large field of view (FOV) by single fringe projection based on metasurface and solve the accurate and robust calibration problem with the depth uncertainty of 4 μm. With a compact metasurface projector, the demonstrated method can achieve submillimeter positioning accuracy under the FOV of 88°, offering robust and fast 3D reconstruction of the texture-less scene due to the modulation characteristic of the fringe. Such scheme may accelerate prosperous engineering applications with the continued growth of flat-optics manufacturing process by using metadevices.

## 1 Introduction

Optical 3D sensors play a key role in the booming 3D information technique, which are ubiquitous in industry [1–3], medicine [4], artistry [5, 6], and virtual reality [7]. And compact size and high performance optical devices are always the ultimate goals to pursue [8]. Recently, combined with the computer vision technique, metasurface [9, 10] enables the miniaturization of optical devices, endowed with flexible arrangement in the application scene with low cost and complexity. Metasurface is an artificial optical surface composed of subwavelength scale two-dimensional array, providing novel modulation of amplitude [11, 12], phase [13, 14], polarization [15, 16] and orbital angular momentum [17, 18] of light field. With the advantage of compact size and large numerical aperture [19], metasurface has already been introduced in some innovative applications of holography display, beam shaping, and optical computing, which surpass the performances of conventional lenses and diffractive optical elements (DOE) [20, 21].

Prior metasurface-based 3D sensors generally belong to passive imaging technique, which enable the miniaturization of 3D sensors and the achievement of 3D positioning or imaging. For example, both the bifocus metalens [22] and points spread function (PSF)-engineered metasurface [23] are based on designed depth-dependent response of metasurface. Inspired by depth from defocus (DFD), the bifocus metalens simultaneously capture two differently defocused images for depth extraction. As the double-helix beam generated by phase-coded metasurface rotates with the depth, the depth information is retrieved by the captured image including two identical scenes with a rotation angle. In addition, light field-based 3D imaging technique such as light-field camera [24] and metalens array [25] have been proposed. Both they can obtain the depth information because of multiple viewpoints by using the lens array, which is akin to multi-view 3D reconstruction. Since they rely the image capture of the multiple viewpoints or multiple modulated depths, these 3D imaging techniques inherit the limitation of the tradeoff between spatial information and depth information. Recently, some active 3D imaging methods based on dots projection [8, 26] are proposed. The large space-bandwidth product of metasurface offers the high density dots projection which can reach to 10 k∼20 k, showing the potential solution of active imaging method on the improvement of the spatial resolution. But the depth reconstruction is time-consuming since the calculation involves a large number of matching operations.

However, metasurface can be introduced in the 3D imaging system as a coded light source to offer another information freedom, which brings extra advantages of tackling the 3D imaging problem of texture-less object and working in the dark conditions. Therefore, we have studied the active 3D positioning and imaging based on metasurface-based device. Here, we propose a single fringe illumination modulation to achieve the frequency coding of the captured image, which is robust for single-shot depth deduce. The geometric metasurface is used for projecting the designed fringe and the deformed fringe image captured by the reflection of the object can be used to reconstruct the depth information. Considering the phase ambiguity caused by the single-direction fringe and phase unwrapping, we propose a calibration model by introducing the epipolar constraint and similarity search, and the corresponding algorithm with alternating direction method of multipliers (ADMM), resulting a re-projection error of 0.2 pixel. Meanwhile, based on the calibration model, the 3D positioning algorithm is proposed and experimentally demonstrated with the positioning accuracy of 0.5 mm in the working distance ranging from 300 mm to 400 mm. Finally, we conduct a 3D facial imaging experiment successfully, indicating the practicability of proposed method in the real scene. In addition, in conjunction with scalability to light source integrating [27], we believe that our method will accelerate the application in computer vision, personal authentication and artificial intelligence et al. with the drastically improved compactness and robust 3D positioning and imaging performance.

## 2 Results

**Figure 1:**
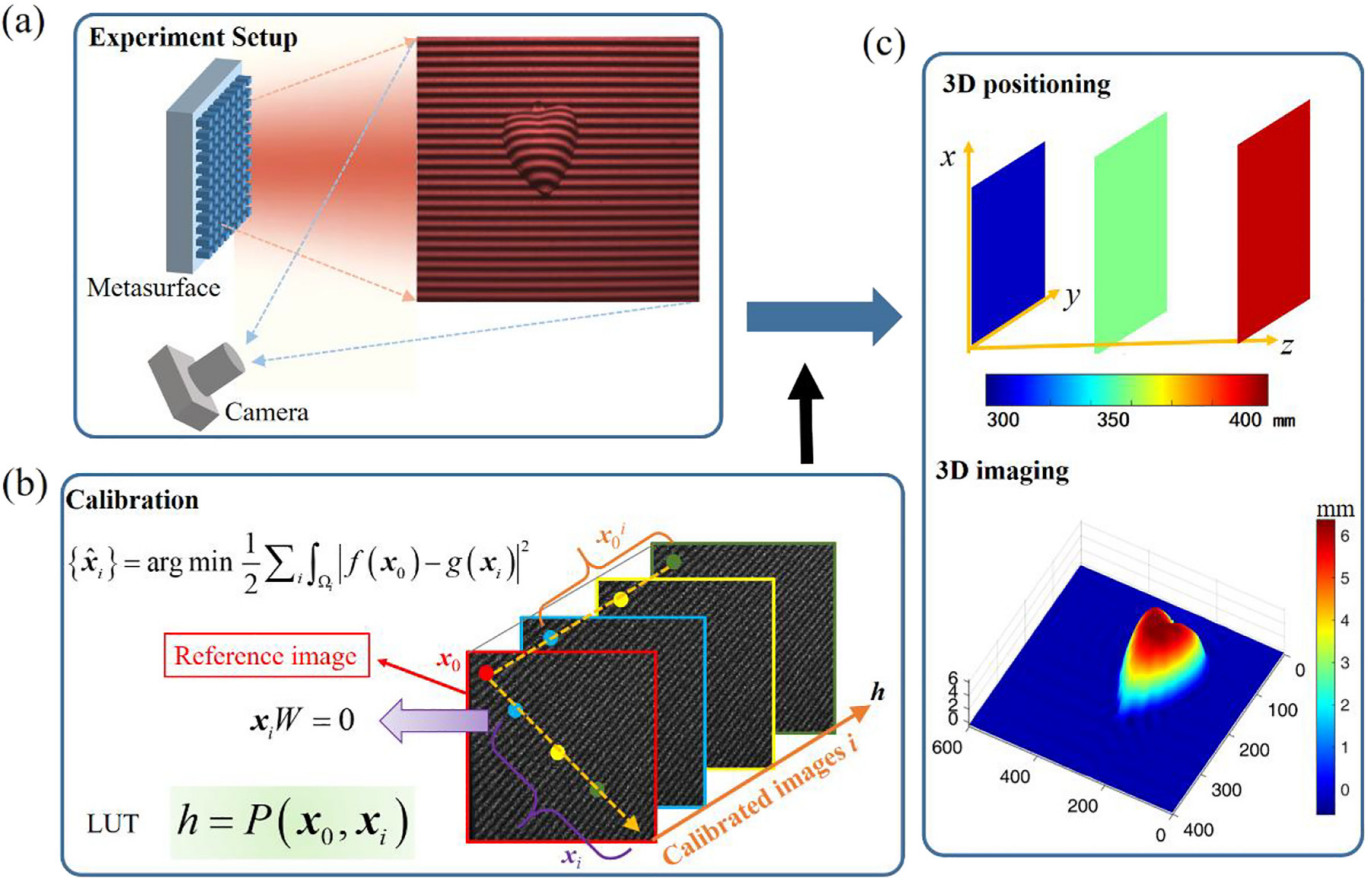
Schematic of proposed method and principle of operation. (a) Experiment setup for 3D imaging system. (b) Calibration algorithm. The loss function of optimization algorithm is similarity evaluation, and the line constraint ***x***_*i*_*W* = 0 denotes that the corresponding points ***x***_*i*_ of the certain pixel position ***x***_*0*_^*i*^ (red, blue, yellow, and green point in each calibrated image *i*) should be determined along a certain line (red, blue, yellow, and green point in the reference image) in the reference image. (c) Application of 3D positioning and imaging.

Active 3D imaging can cope well with the texture-less objects that frequently encountered in the real scene, because the coding illumination offers extra information for depth estimation. Here we proposed a metasurface device for single-shot 3D positioning and imaging as shown in Figure 1. The fringe projection is used here for robust depth extraction, leveraging the advantage of effective noise removal as a kind of encoding in Fourier space. Combined with the triangulation, depth information can be calculated by deformed fringe image, which is captured by the camera from the reflection of the object as shown in Figure 1a.

Note the single illumination fringe cannot describe the spatial position without ambiguity because of the single-directional encoding characteristic, which yields some sticky problems of accurate calibration. Assuming the fixed pixel position ***x***_*0*_^*i*^ of the *i*th calibration image has the corresponding points ***x***_*i*_ (in the reference image) as shown in Figure 1b, we solve the sub-pixel matching problem with finely designed algorithms combined geometrical constraint with the similarity requirement of ***x***_*i*_. Thus the relation **P** between the depth *h* and the correspondence (***x***_*0*_, ***x***_*i*_) can be obtained by proposed optimization algorithm leveraging the nonlinear mathematical relation between them based on triangular geometry (see the details in the look-up table of Supplementary Note 4) metrology method. Herein, we propose a fast, accurate positioning method based on the priori calibration results **P**, which can also be applied in 3D imaging shown in Figure 1c.

**Figure 2:**
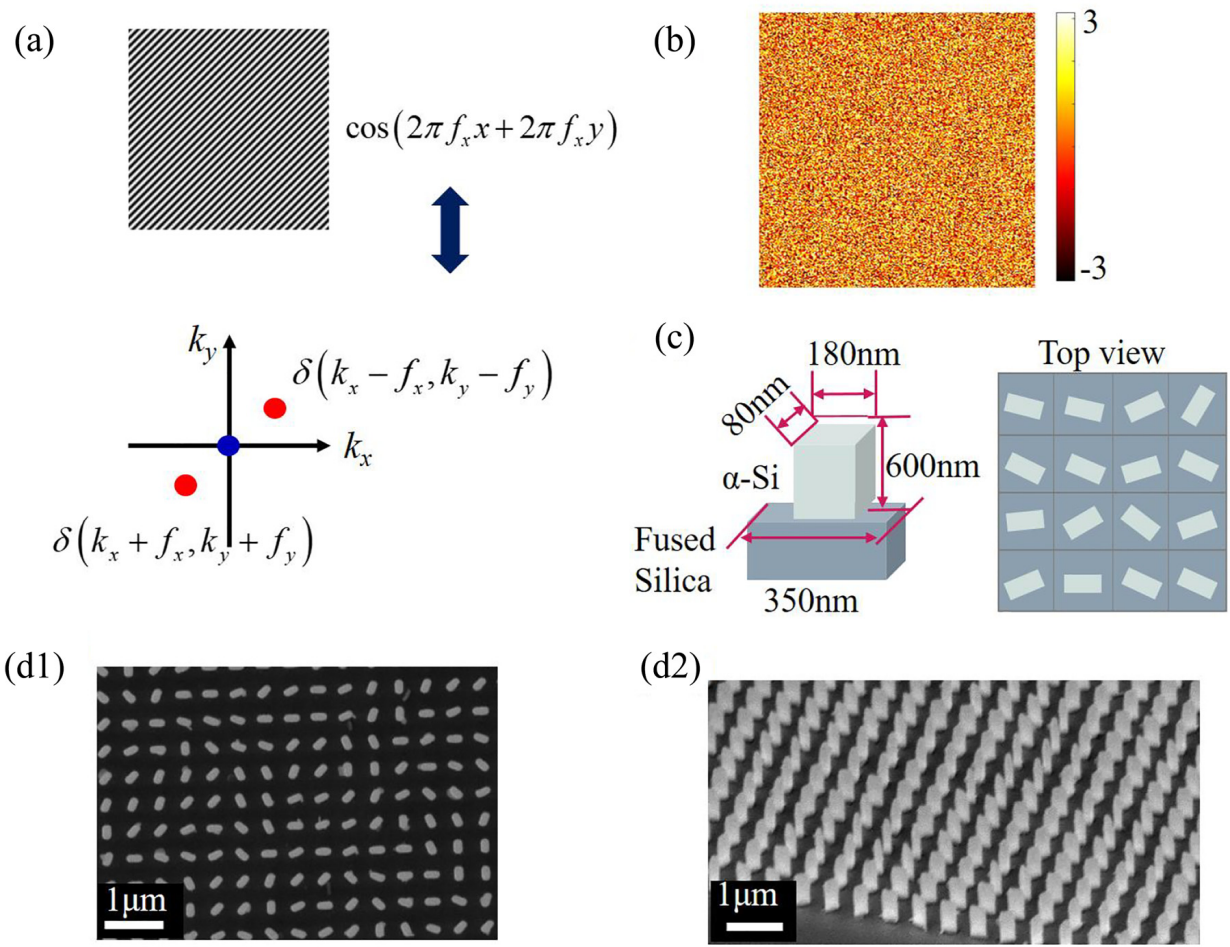
Metasurface design. (a) Functionality of fringe modulation. Depth information is embedded in the phase around the frequency component of modulated fringe. (b) Phase profile provided by metasurface. (c) Nanofin design. (d) SEM images. d1 and d2 are the top view and side view, respectively.

The captured image can be modeled as a deformed fringe modulated by the depth information as shown in Figure 1a, and we represent this as follows,

(1) $$I\left(x,y\right)=A\left(x,y\right)+B\left(x,y\right)\cos\left[2\pi f_{x}x+2\pi f_{y}y+\varphi \left(x,y\right)\right]$$

where *I*(*x*, *y*) is the intensity distribution of the captured image, and *A*(*x*, *y*) and *B*(*x*, *y*) are the background intensity and object reflectivity, respectively. The undeformed fringe with the spatial frequency *f*_*x*_, *f*_*y*_ equaling to 1/10 pixel^−1^ is shown in Figure 2a. The captured image reflected from the object is regarded as a deformed fringe added a phase term *φ*(*x*, *y*) nonlinearly related to the depth *h*(*x*, *y*) in the original fringe, which the nonlinear relationship depends on the system parameters including the relative position between the metasurface and camera, and the focal length of camera. Leveraging the even pulse of cosine function as shown in Figure 2a, we transform *I*(*x*, *y*) in Fourier space to describe the spectrum $\tilde{I}\left(k_{x},k_{y}\right)$ ,

(2) $$\begin{array}{ll} \tilde{I}\left(k_{x},k_{y}\right) & =\tilde{A}\left(k_{x},k_{y}\right)+Q\left(k_{x}-f_{x},k_{y}-f_{y}\right)\\ & \;+Q^{*}\left(k_{x}+f_{x},k_{y}+f_{y}\right) \end{array}$$

where *Q* is the Fourier spectrum of $b\left(x,y\right)\exp\left[j\varphi \left(x,y\right)\right]/2$ , and Q* is the conjugate of *Q*. As we can see, the fundamental frequency component *Q* carries the phase information *φ*(*x*, *y*), and is separable from zero-order component $\tilde{A}\left(k_{x},k_{y}\right)$ , which is the Fourier spectrum of the background intensity *A*(*x*, *y*). Therefore, the depth estimation can be achieved by the phase extraction calculation based on frequency filtering and inverse Fourier transform (see Supplementary Note 1). For the high efficiency of holographic reconstruction, we design the phase hologram as shown in Figure 2b, which is calculated by Gerchberg–Saxton (GS) algorithm with angle of full FOV (field of view) equaling to 88°.

We choose the meta-structure made of amorphous silicon to implement the phase engineering. By leveraging the advantage of broadband performance and robustness against fabrication errors of geometric metasurface, amorphous silicon nanopillars with different orientation angles *θ* (*x*, *y*) are arranged on a fused silica substrate to fulfill the desired phase profile. The output electric field *E*_out_ transmitted through the nanopillars can be described as

(3) $$E_{\text{out}}=\frac{t_{l}+t_{s}}{2}\left[\begin{array}{c} 1\\ \mp i \end{array}\right]+\frac{t_{l}-t_{s}}{2}e^{\pm i2\theta \left(x,y\right)}\left[\begin{array}{c} 1\\ \pm i \end{array}\right],$$

where *t*_*l*_ and *t*_*s*_ denote the complex transmission coefficients corresponding to the long and short axis of the rectangular nanopillars. Therefore, ${\left|\frac{t_{l}-t_{s}}{2}\right|}^{2}$ is required for high polarization conversion efficiency by optimizing the structure parameters such as height, length, and width of the nanopillars. The period and height of nanopillars are chosen as 316 nm and 600 nm, respectively for covering the phase shifts from 0 to 2*π*. A rigorous coupled wave analysis (RCWA) method is used for optimizing the 2D parameter of nanopillar at the operating wavelength of 633 nm and the optimized length and width are determined as 180 nm and 80 nm (see the efficiency analysis in Supplementary Note 2). Our fabricated metasurface is composed of 1578 × 1578 nanopillars by using electron beam lithography and reactive ion etching, and the corresponding scanning electron microscopy images with a top view and side view are shown in Figure 2d.

**Figure 3:**
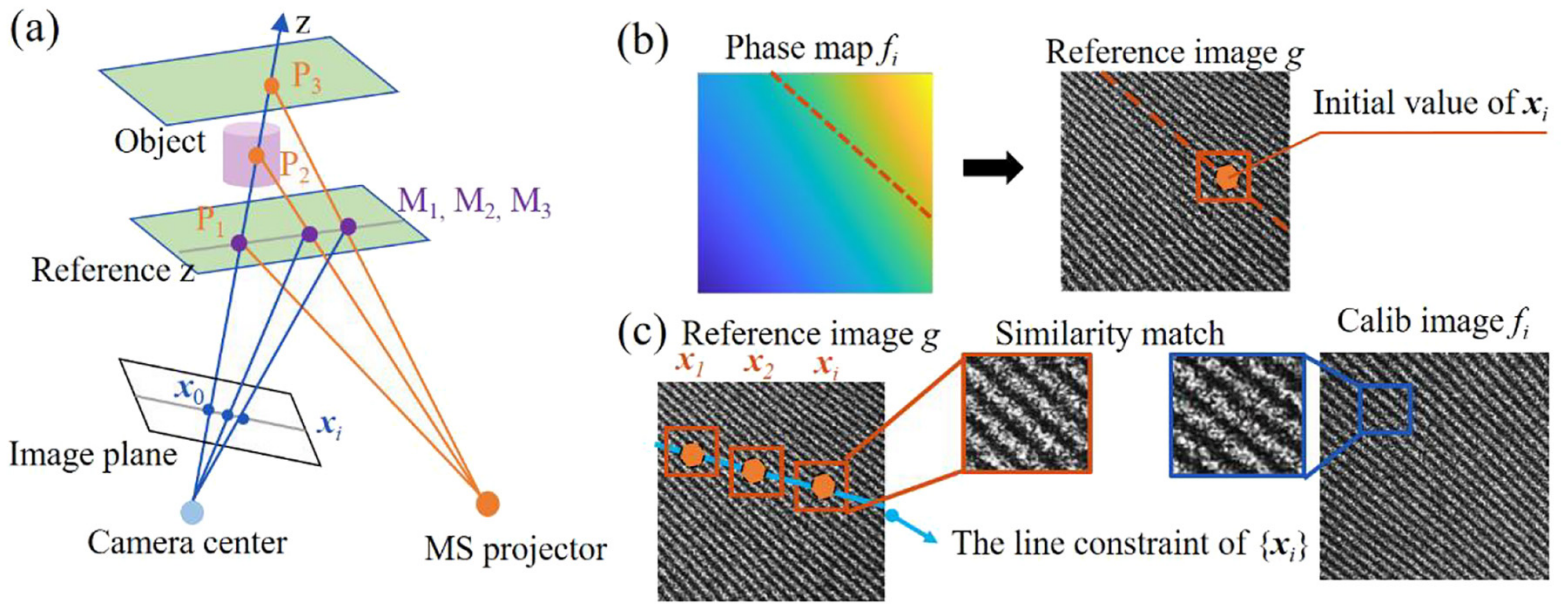
Calibration algorithm and results. (a) Epipolar geometry in the 3D imaging system. (b) The initial value of ***x***_*i*_. The initial corresponding coordinate is estimated by finding the most similar points from the regions with proximal phase value in reference image. The orange line denotes the regions with proximal phase. (c) The goal of the calibration algorithm. The calibration aims to make each point ***x***_*i*_ have a high similarity with the corresponding calibrated image *f*_*i*_ and stands in a line.

The fringe modulation conducts the search of ***x***_*i*_ by determining the extracted phase equaling to the phase of ***x***_*i*_^0^, but the single-directional fringe results the indistinguishable points along the direction of fringe which have the identical phase. Note the key problem in the triangulation is the determination of the corresponding points; we introduce the epipolar geometry [28] as search constraint, which is described in Figure 3a. The spatial points set {*P*_1_, *P*_2_, *P*_3_} imaged at the certain pixel position ***x***_0_ of image plane is captured in different depths, and the corresponding projection pattern is same to the one of points set {*M*_1_, *M*_2_, *M*_3_} in reference plane. According to the geometric characteristic, the points in set {*M*_1_, *M*_2_, *M*_3_} keep in a line, and the corresponding points {***x***_*0*_, ***x***_*i*_} in the image plane also meet the requirement of collinearity. As we can see in Figure 3b, the captured projection pattern has some speckles, which keeps a certain degree of similarity among the calibrated images (The experiment validation can be seen in Supplementary Note 3). Consequently, for a given set of calibrated images, the problem of searching the corresponding points is recast to finding the most similar points {***x***_*i*_} to the certain point ***x***_0_, and they can precisely fall on one straight line at the same time. The correspondence matching is modeled as

(4) $$\begin{array}{cc} \left\{\hat{x},\hat{y}\right\} & =\mathrm{arg min}\frac{1}{2}\underset{i}{\sum }\underset{\Omega_{i}}{\int }\left|f_{i}\left(x_{0}+\Delta x,y_{0}+\Delta y\right)\right.\\ & \;{\left.-g\left(x+\Delta x,y+\Delta y\right)\right|}^{2}\;i=2:N\\ st\;uW & =0,u=C\left(x,y\right) \end{array}$$

where $\left\{x,y\right\}$ is the spatial coordinate of optimized corresponding points set {***x***_*i*_} of *N* calibrated images, and *u* is the matrix transformed from (*x*, *y*) by the operation **C**. *W* is the auxiliary matrix for line constraint (see the details in Supplementary Note 4). *f*_*i*_, *g* are the calibrated image with the serial number *i* and the reference image, respectively. ∆*x*, and ∆*y* are used to assure that the calculated points are in the sub-region Ω_*i*_. The initial value of ***x***_*i*_ can be estimated by the proximal phase search and similarity determination.

The phase distribution of each calibrated image can be calculated by frequency filter and inverse Fourier transform (see Supplementary Note 1), then we can find the possible positions according to the proximal phase value of *φ*_0_(*x*_0_, *y*_0_) in the reference image, which is shown as an orange line in Figure 3b. The initial estimation of ***x***_*i*_ can be determined by finding the most similar point in the orange line. Based on the initial estimation, we develop the algorithm solver of Eq. (3) with alternating direction method of multipliers (ADMM) [29]. One of the optimization directions is similarity match between the{***x***_*i*_} and the corresponding calibrated points ***x***_*i*_ as shown in Figure 3c, and the other is the line constraint of {***x***_*i*_} (see Supplementary Note 4). Meanwhile, we design an iteration method based on the least squares on the basis of the relation between depth *h* and spatial coordinate $\left(\hat{x},\hat{y}\right)$ in the image plane,

(5) $$h\left(x,y\right)=P\left(\hat{x},\hat{y};a,b\right)$$

where **P** denotes the mathematical operator (see Supplementary Note 4), and *a*, *b* are related to system parameters such as the relative position between the metasurface and camera, and the focal length of camera, thus the look-up table (*a*, *b*) can be established for fast 3D reconstruction.

**Figure 4:**
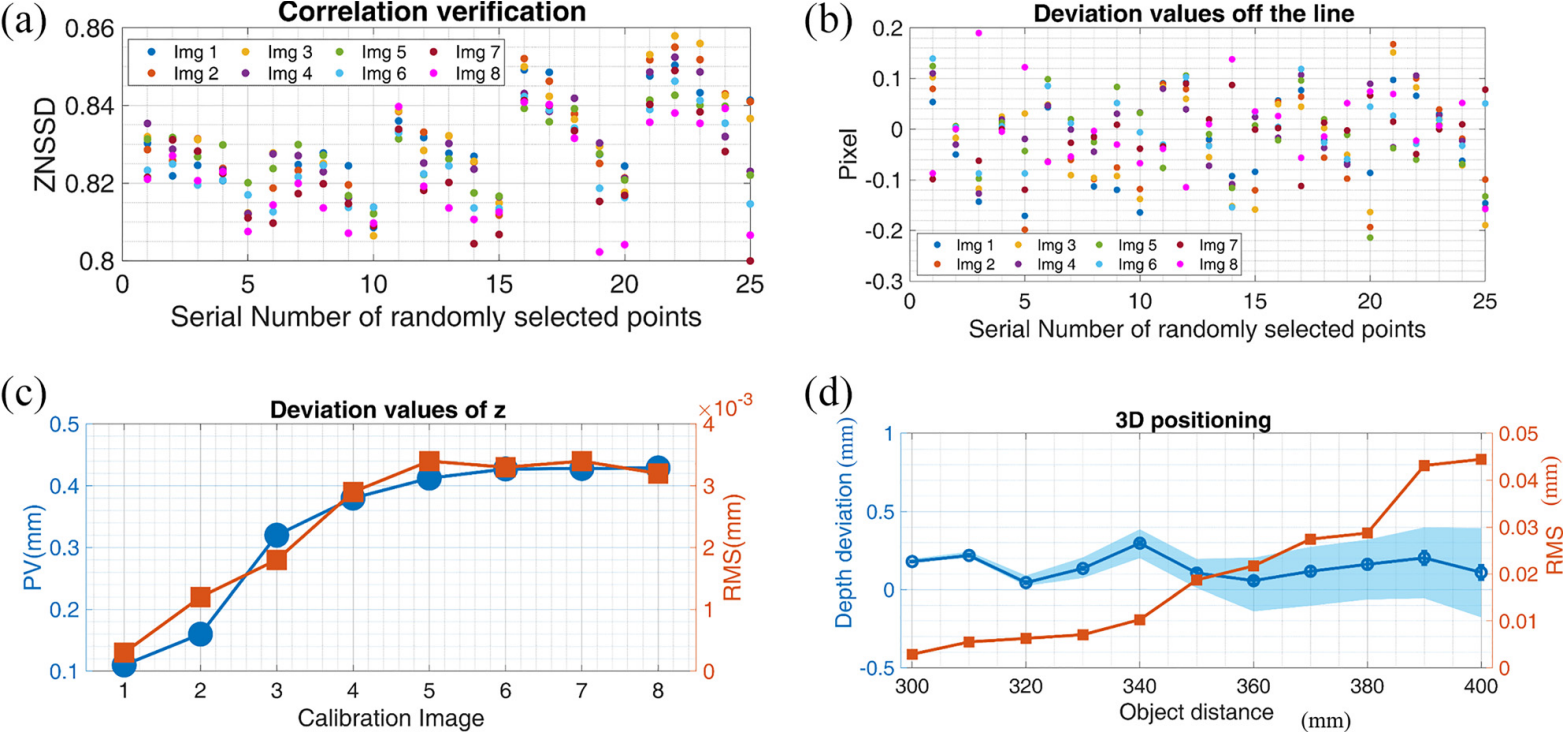
Calibration results and 3D positioning results. (a) Correlation values ZNSSD obtained by the optimized corresponding points. (b) Deviation values off the line calculated by the optimized corresponding points. (c) PV and RMS value calculated by the optimized corresponding points and look-up table. (d) Positioning results. The blue line represents the deviation values between the average value of reconstructed depth and the real depth value, and the bluish area is bounded by the maximum and minimal deviation of the depth distribution in each experiment. The orange line represents the RMS (root-mean-square) value of the depth distribution in each experiment.

To demonstrate the validity of calibration results, the correlation values zero-normalized sum of squared difference coefficient (ZNSSD) and deviation off the line are calculated as shown in Figure 4a and b, respectively, which can be used to evaluate the correctness of the corresponding points directly.

(6) $$\begin{array}{ll} C_{\text{ZNSSD}} & =\overset{M}{\underset{x=1}{\sum }}\overset{N}{\underset{y=1}{\sum }}\left[\frac{f\left(x,y\right)-f_{m}}{\sqrt{\overset{M}{\underset{x=1}{\sum }}\overset{N}{\underset{y=1}{\sum }}{\left[f\left(x,y\right)-f_{m}\right]}^{2}}}\right.\\ & \;{\left.-\frac{g\left(x^{\prime },y^{\prime }\right)-g_{m}}{\sqrt{\overset{M}{\underset{x=1}{\sum }}\overset{N}{\underset{y=1}{\sum }}{\left[g\left(x^{\prime },y^{\prime }\right)-g_{m}\right]}^{2}}}\right]}^{2} \end{array}$$

The results are calculated by 25 randomly selected points in the selected calibrated image named Img 1, Img 2, …, Img 8. As we can see, all the correlation values are greater than 0.8, and the absolute values of deviation are smaller than 0.2 pixel. Therefore, the optimized corresponding points obtained by the proposed algorithm are proven to fulfill with the requirement of similarity and epipolar constraint. We also use the optimized corresponding points and look-up table to calculate the depth value *h* (*x*, *y*) of each calibrated image, and the peak valley (PV) and root mean square (RMS) value of each depth value *h* (*x*, *y*) are shown in Figure 4c. The PV value is smaller than 0.5 mm and RMS value is smaller than 4 × 10^−3^ mm, showing a sub-millmeter calibration accuracy.

Therefore, we design an accurate positioning algorithm based on the similarity match, epipolar constraint and look-up table. Firstly, the wrapped phase is obtained by phase extraction calculation (see Supplementary Note 1) and a global initial search method is used to unwrap the phase image for absolute phase (see Supplementary Note 5). Combined with the epipolar constraint, the absolute phase image can be used to determine a rough area for searching the corresponding points. Thus the subpixel correspondence can be obtained with the correspondence matching algorithm (see Supplementary Note 4), resulting in an accurate depth estimation by using the look-up-table (*a*, *b*). To quantify the positioning accuracy, we design the positioning experiment by 11 groups of object distances ranging from 300 mm to 400 mm apart from the metasurface. As shown in Figure 4d, the reconstructed depths are in relatively good agreement with the real object distance, which is obtained by modulating precise translation stage. The reconstructed depth deviations are smaller than 0.5 mm, owing to the proposed positioning algorithm (see the comparison with traditional Fourier transform profilometry [30] in Supplementary Note 5) and accurate calibration data which has been proven in Figure 4c. Note the depth error increases with the increasing object distance, the main reason is the decorrelation effect (see Supplementary Note 3) with the increasing relative depth based on the reference plane.

**Figure 5:**
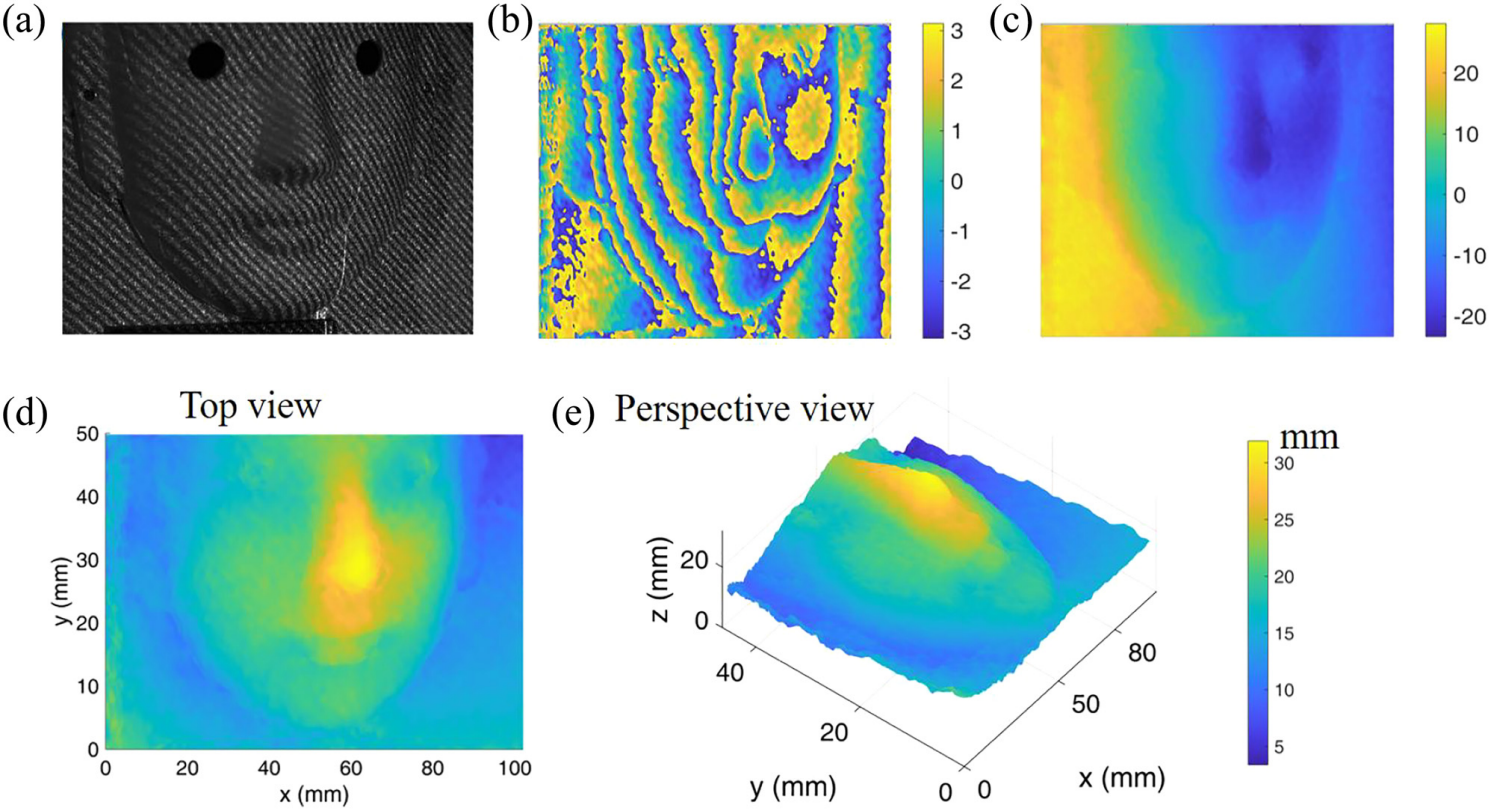
3D facial imaging. (a) Captured image. (b) Wrapped phase. (c) Unwrapped phase. (d) Top view of 3D image. (e) Perspective view of 3D image.

In addition to the positioning technique, we also demonstrate the ability of 3D imaging reconstruction as shown in Figure 5. The deformed unidirectional fringe image of a facial mask is captured by a camera, indicating that the nose with a relatively high depth makes the projection fringe a sharp twist shown in Figure 5a. Thus the phase information of the fringe can be obtained clearly and fast by the phase extraction method and global initial search shown in Figure 5b and c, which offers rough but robust corresponding points estimation with the reference image. The 3D result of the facial mask is completely reconstructed as shown in Figure 5d and e, which can be attributed to the accurate look-up table (*a*, *b*) computed by our proposed calibrated algorithm for the transformation between the depth and the correspondence matching. Note that the corresponding points search of the facial mask is operated by phase information and epipolar constraint without similarity matching, since the decorrelation effect occurs in the discrepant surface. Therefore, our proposed method can achieve fast (computing time is smaller than 0.05 s) and robust 3D imaging.

## 3 Conclusions

In summary, an active 3D positioning and imaging method with compact metasurface is proposed in this work, which benefits from the flexible light field control ability of metasurface and elaborate calibration and reconstruction algorithm. Note that previous metasurface-based passive imaging such as metalens array generally needs to capture multi-views in a single detector, the depth information is obtained with the sacrifice of the spatial information. Leveraging the extra information offered by the metasurface–based illumination, our proposed method not only takes advantage of the active imaging to achieve the 3D imaging without the waste of the spatial information, but also demonstrate a coding illumination application examples as high-performance 3D positioning and imaging implementation with advanced metasurface-based optical systems, offering another design freedom for the growing metasurface-based imaging systems. Furthermore, we develop a complete technical route for active 3D imaging with metasurface-based device. We establish the calibration model and the corresponding calibration algorithm including the algorithm of correspondence matching and look-up table by leveraging the geometric relation of system and the pattern characteristic sufficiently. The calibration results demonstrate the validity of proposed calibration method experimentally, laying the foundation of accurate 3D positioning. Eventually, the quantitative positioning accuracy is provided, and the 3D facial imaging is achieved successfully, indicating that the proposed method can be applied in the real scenario. Note that decorrelation effect may harm the spatial resolution of 3D imaging, multi-periodic [31] or phase-shifting fringe [32] projection fringe projection based on multiplexing technique of metasurface [15, 33] has the potential for 3D reconstruction of high spatial resolution. We believe that the proposed method is an elegant integration of nanophotonic technology with computational photography in the field of active imaging, which may inspire the further development of endoscopy vision, industrial inspection, and automatic vehicle.

See Supplementary Material for the complete algorithm derivation.

## Supplementary Material

Supplementary Material Details
