# Supplementary material for: Active 3D positioning and imaging modulated by single fringe projection with compact metasurface device
Source: Nanophotonics. 2023 Apr 4;12(10):1923–30. doi: 10.1515/nanoph-2023-0112 (PMC11501738; doi:10.1515/nanoph-2023-0112)
Supplement: Supplementary file 1 — Supplementary Material Details [file j_nanoph-2023-0112_suppl_001.pdf]

## Supplementary Note 1: Phase extraction calculation

We named the cropped spectrum of $\tilde{Q}$, $\tilde{Q}^*$ as $\tilde{Q}_c$, $\tilde{Q}_c^*$, respectively. The frequency filtering is conducted by selecting the close region around the fundamental frequency $f_x$ in the spectrum of the image $\tilde{I}$, which is shown as the dashed rectangular box in Fig. S1(a). Then the inverse Fourier transform of $\tilde{Q}_c$, $\tilde{Q}_c^*$ is calculated:

$$q_c = IFT(Q_c) \tag{S1}$$

where $q_c$ corresponds to $b(x,y)\bullet\exp[j\varphi(x,y)]/2$. Then the phase $\varphi(x, y)$ can be determined by extracting the phase of $q_c$, which is the inverse Fourier transform of $Q_c$.

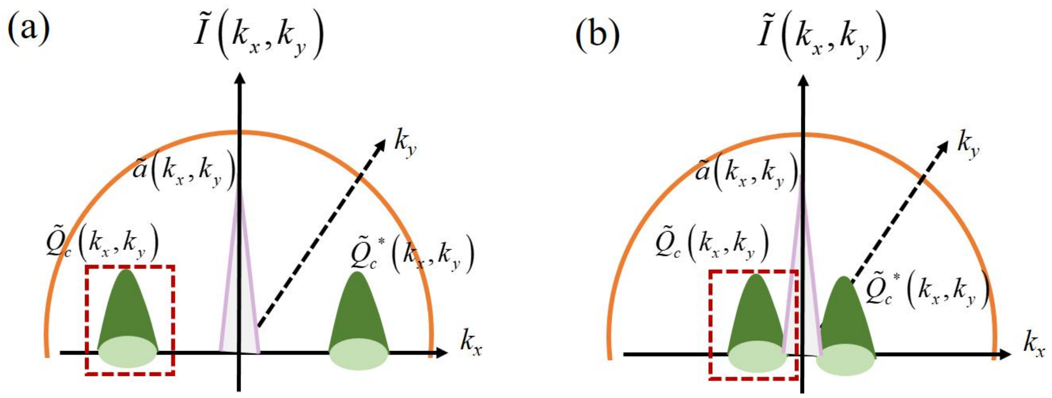


Fig.S1 The phase extraction without (a) and with (b) spectrum overlapping.

The geometric parameters of fringe projection are shown in Fig. S2. The pitch is the minimum period of the whole fringe pattern, and the width represents the width of the black or white fringe. Since the intensity of fringe pattern satisfies the cosine function as shown in Eq. (1), the pitch and the width is $1/f_x$ and $1/(2\,f_x)$, respectively. There are two main considerations in the setting of geometric parameters of fringe. One is the imaging resolution, and the other is quality of the holographic projection.

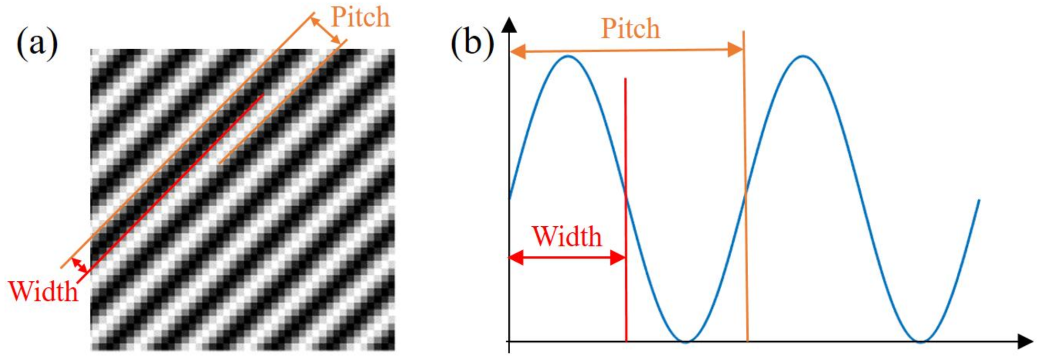

Fig.S2 The diagram of geometric parameters in fringe projection.

The imaging resolution is limited by the quality of the phase extraction, which may become blurred due to spectrum overlapping. As shown in Fig.S1 (b), the fringe with the lower spatial frequency $f_x$ makes it difficult to filter out the -1 order from the zero order, precluding high-accuracy phase calculation of the object. The orange semicircle means the highest frequency determined by the camera as $\beta/2pix$, where $\beta$ and *pix* is the magnification and pixel pitch of the camera. Thus the spatial frequency of the fringe should be set as $max(f_x < \beta/2pix)$.

We use the Gerchberg-Saxton (GS) algorithm to design the phase distribution of metasurface. Considering the quality of holographic reconstruction, our designed pitch of the projection fringe is 10 pixel. The high-quality thinner fringe can be obtained if we use a large-scale metasurface. Therefore, the imaging resolution will be improved through an advanced micro-nano fabrication.

## Supplementary Note 2: Metasurface design

The simulated results of the transmission coefficient of polarization conversion efficiency are shown in Fig. S3. We have used amorphous silicon with refractive index ($n$=4.1528) and extinction coefficient (k=0.08) for validation. The length and width are determined as 180 nm and 80 nm for maintaining high transmission efficiency. The simulated and measured polarization conversion efficiency at the wavelength of 633 nm is 52% and 20%, respectively. By engineering planar nanostructures, the desired phase profile was then converted into a diverse orientation distribution based on PB phase principle.

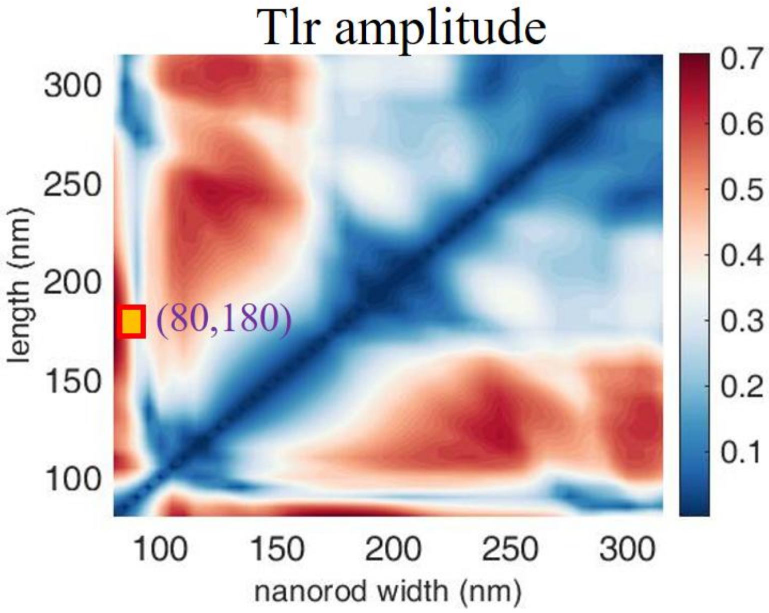


Fig. S3 Transmission coefficients by sweeping the geometric parameters of nanopillar within a unit cell.

## Supplementary Note 3: Similarity Validation

We have captured 11 groups of images with depth interval 5 mm ranging from 300 mm to 350mm away from the metasurface for calibration, while the measurement setup keeps constant. Then we used ZNSSD for evaluation of the similarity. Firstly, we use two randomly selected calibrated images for calculating the correlation of the local region, which is shown as a red rectangular box in Fig. S4 (a). The enlarged views show a good similarity with the ZNSSD value 0.78. Then, for statistical purpose, the correlation of all the calibrated images are calculated, and the average value of each image is shown in Fig. S4 (b), indicating that the calibrated images have the ZNSSD coefficients greater than 0.7. Note that the ZNSSD values decreasing with the increasing distance away from the reference image, the decorrelation effect occurs in the axial distance of the metasurface.

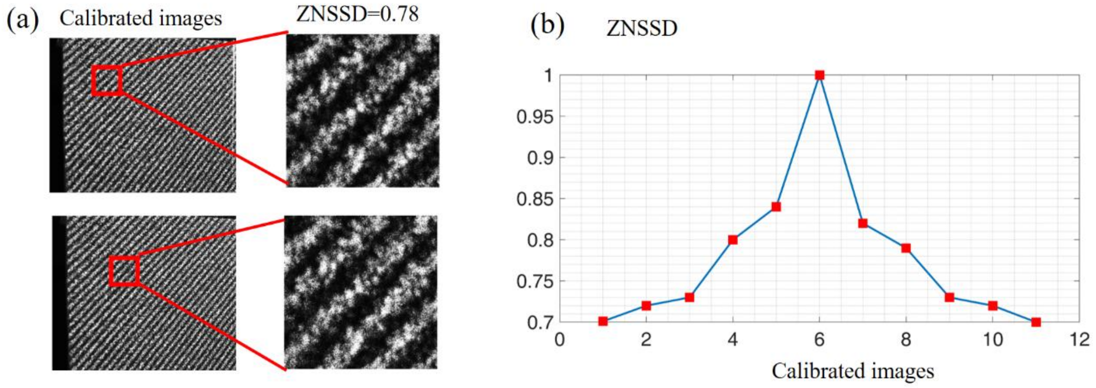


Fig. S4 Correlation of the fringe pattern in various depth. (a) Local correlation of randomly selected calibrated images. The images are cropped for display. (b) Correlation of different depths. The 6$^{th}$ image is the reference image.

## Supplementary Note 4: Calibration algorithm

### A. Derivation of ADMM solver for correspondence matching

We use the alternating direction method of multipliers (ADMM) to solve Eq. 3 in the manuscript by designing new variables. Then, Eq. 3 can be recast as:

$$\begin{aligned}&\{\Delta\hat{\boldsymbol{p}}\} = \arg\min \frac{1}{2}\sum_i \int_{\Omega_i} \left| f_i(\boldsymbol{x}_0 + \Delta\boldsymbol{x}) - g\left(\boldsymbol{x}_i + \Delta\boldsymbol{x} + \nabla g \frac{\partial S}{\partial \boldsymbol{p}_i} \bullet \Delta\boldsymbol{p}_i\right)\right|^2 \\ &st \quad uW = 0, u = \mathrm{C}(\boldsymbol{x}_i + \frac{\partial S}{\partial \boldsymbol{p}_i} \bullet \Delta\boldsymbol{p}_i)\end{aligned} \tag{S2}$$

where $\boldsymbol{x}_i = [x_i, y_i]^{\mathrm{T}}$ and $\Delta\boldsymbol{x} = [\Delta x, \Delta y]^{\mathrm{T}}$. ($\Delta x$, $\Delta y$) is the pixel shift between the center pixel $\boldsymbol{x}_i$ and the neighbor pixel in the calculated region $\Omega_i$, then the first term $f_i(\boldsymbol{x}_0 + \Delta\boldsymbol{x})$ denotes the image in the region $\Omega_i$ and $g$ denotes the reference image. The second term denotes the corresponding region needed to be optimized. We introduce shape function $S$ ($\boldsymbol{x}_0$; $\boldsymbol{p}$) to describe the deformation as follows.

$$S(\boldsymbol{x}_0; \boldsymbol{p}) = \begin{bmatrix} 1+u_x & u_y & u_0 \\ v_x & 1+v_y & v_0 \\ 0 & 0 & 1 \end{bmatrix} \begin{bmatrix} \Delta x \\ \Delta y \\ 1 \end{bmatrix} \tag{S3}$$

$$\boldsymbol{p} = (u_0, u_x, u_y, v_0, v_x, v_y)^T \tag{S4}$$

Meanwhile, $\Delta\boldsymbol{p}$ is the increment of $\boldsymbol{p}$ as:

$$\Delta\boldsymbol{p} = (\Delta u_0, \Delta u_x, \Delta u_y, \Delta v_0, \Delta v_x, \Delta v_y)^T \tag{S5}$$

The constraint term denotes that the corresponding points for each calibrated image should fall in a certain line. The matrix variables $W$ is the matrix with the certain line equation,

$$W = \begin{bmatrix} 1 & 0 \\ 0 & 0 \\ 0 & 1 \end{bmatrix} \left([u_x, I_{N\times 1}]^{\mathrm{T}} [u_x, I_{N\times 1}]\right)^{-1} [u_x, I_{N\times 1}]^{\mathrm{T}} u_y + \begin{bmatrix} 0 \\ -1 \\ 0 \end{bmatrix}$$

(S6)

where $I_{N\times 1}$ is a matrix of size $N\times 1$ and every element is 1. Then $uW$=0 denotes that the distance from all the points to the certain line should be zero. The operator C is used to reshape $\boldsymbol{x}_i$ to $u$,

$$u = \left( \boldsymbol{x}_i + \frac{\partial S}{\partial \boldsymbol{p}_i} \bullet \Delta \boldsymbol{p}_i \right) \quad C$$

$$\downarrow \qquad \downarrow \qquad \downarrow \tag{S7}$$

$$\overbrace{\left[ u_x \quad u_y \quad I^{N\times1} \right]} = \overbrace{\left[ \tilde{x}_i \quad \tilde{y}_i \right]} \quad \overbrace{\begin{bmatrix} 1 & 0 & 0 \\ 0 & 1 & 0 \end{bmatrix} + \left[ 0 \quad 0 \quad I^{N\times1} \right]}$$

Augmented Lagrangian arguments can be written,

$$L = \frac{1}{2} \sum_i \int_{\Omega_i} \left| f(\boldsymbol{x}_0) - g\left( \boldsymbol{x}_i + \frac{\partial S}{\partial \boldsymbol{p}} \bullet \Delta \boldsymbol{p} \right) \right|^2 + \frac{\lambda_1}{2} \| u\mathrm{W} + \xi_1 \|_2^2 + \frac{\lambda_2}{2} \left\| u - C(\boldsymbol{x}_i + \frac{\partial S}{\partial \boldsymbol{p}} \bullet \Delta \boldsymbol{p}) + \xi_2 \right\|_2^2 \tag{S8}$$

Using ADMM, we break up Eq. S9 into three simpler problems. Then we show it at the iteration $k$ as follows.

Subproblem 1: While holding $u$, $\xi_1$, $\xi_2$ fixed, minimize $L$ over $\Delta \boldsymbol{p}^k$, to obtain $\Delta \boldsymbol{p}^{k+1}$:

$$\Delta \boldsymbol{p}^{k+1} = -\left[ H + \lambda_2 \left( \frac{\partial S^T}{\partial \boldsymbol{p}} \frac{\partial S}{\partial \boldsymbol{p}} \right) \right] \times \left[ \left( \nabla g \frac{\partial S}{\partial \boldsymbol{p}} \right)^T \times \left( f - \frac{\Delta f}{\Delta g} \right) g + \lambda_2 \frac{\partial S^T}{\partial \boldsymbol{p}} \left( C^{-1} u^k - X_i^k + \xi_2{}^k \right) \right] \tag{S9}$$

Where $H = \left( \nabla g \frac{\partial S}{\partial p} \right)^T \left( \nabla g \frac{\partial S}{\partial p} \right)$.

Subproblem 2: While holding $\Delta \boldsymbol{p}^{k+1}$, $\xi_1$, $\xi_2$ fixed, minimize $L$ over $u^k$, to obtain $u^{k+1}$:

$$u^{k+1} = \left[ -\lambda_1 \left( bI + \xi_1{}^k \right) a^T + \lambda_2 \left( C(x_i + \frac{\partial S}{\partial \boldsymbol{p}} \bullet \Delta \boldsymbol{p}) + \xi_2{}^k \right) \right] \times \left( \lambda_1 a a^T + \lambda_2 I_{3\times3} \right)^{-1} \tag{S10}$$

Where $I_{3\times3} \in R^{3\times3}$ is the identity matrix.

Subproblem 3: While holding $\Delta \boldsymbol{p}^{k+1}$, $u^{k+1}$, $\xi_1$, $\xi_2$ fixed, minimize $L$ over $W^k$, to obtain $W^{k+1}$:

$$W=\begin{bmatrix}-\left[(u_x^{k+1})^{\mathrm{T}}(u_x^{k+1})\right]^{-1}\times\left[(u_x^{k+1})^{\mathrm{T}}W_0^{\mathrm{T}}W^kI-(u_x^{k+1})^{\mathrm{T}}(u_y^{k+1})\right]\\ -1\\ -(I^{\mathrm{T}}I)^{-1}\times I^{\mathrm{T}}\left[u(W^k+W_1)+\xi_1^k\right]\end{bmatrix} \quad \text{(S11)}$$

Where $W_0=\begin{bmatrix}0\\0\\1\end{bmatrix}$, $W_1=\begin{bmatrix}0\\1\\0\end{bmatrix}$.

Then we update dual variables:

$$\begin{aligned}\xi_1^{k+1}&=\xi_1^k+\lambda_1\left(uW\right)\\ \xi_2^{k+1}&=\xi_1^k+\lambda_2\left[u-\mathrm{C}(\boldsymbol{x}_i+\frac{\partial S}{\partial \boldsymbol{p}}\bullet\Delta\boldsymbol{p})\right]\end{aligned} \quad \text{(S12)}$$

## B. Look-up table

Based on the triangulation, the relative depth $h$ (depth away from the reference plane) can be calculated as:

$$h(x,y)=\frac{x-x0}{a(x_0,y_0)-b(x_0,y_0)*(x-x_0)} \quad \text{(S13)}$$

Then we rewrite Eq. S14 as:

$$\begin{bmatrix}(x_1-x_0) & 1\\ (x_2-x_0) & 1\\ \vdots & \vdots\\ (x_N-x_0) & 1\end{bmatrix}\begin{bmatrix}b(x_0,y_0)\\ a(x_0,y_0)\end{bmatrix}=\begin{bmatrix}(x_1-x_0)\\ (x_2-x_0)\\ \vdots\\ (x_N-x_0)\end{bmatrix}\odot 1/h_i \quad i\in(1,N)$$

(S14)

where $\odot$ denotes dot product. Obviously, we can use the pixel coordinates of all the corresponding points $x_i$ for a certain pixel ($x_0$, $y_0$) and the initial depth value $h_i$ obtained by the translation stage to deduce the least square solve of $a$ ($x_0$, $y_0$), $b$ ($x_0$, $y_0$). Eq. S14 can be also written as:

$$\begin{bmatrix}(x_i^1-x_0^1)\odot b(x_0,y_0)-a(x_0,y_0)\\ (x_i^2-x_0^2)\odot b(x_0,y_0)-a(x_0,y_0)\\ \vdots\\ (x_i^M-x_0^M)\odot b(x_0,y_0)-a(x_0,y_0)\end{bmatrix}h_i=\begin{bmatrix}-(x_i^1-x_0^1)\\ -(x_i^2-x_0^2)\\ \vdots\\ -(x_i^M-x_0^M)\end{bmatrix} \quad \text{(S15)}$$

where $m$ denotes the number of all the pixels in the camera plane. Similarly, we can use the pixel coordinates of the corresponding points $x_i$ for a certain calibrated plane

and $a(x_0, y_0)$, $b(x_0, y_0)$ to deduce the least square solve of $h_i$.

Thus we can design iteration schematic for the optimized $a\ (x_0, y_0)$, $b\ (x_0, y_0)$ and $h_i$ by using Eq. S15 and Eq. S16. The pseudocode is shown as follows.

**Algorithm for look-up table**

**Input**: Corresponding points $x_i^j$ and initial depth $h_i^{(0)}$

**Output**: $a\ (x_0, y_0)$, $b\ (x_0, y_0)$ and $h_i$

**While** $\left\| a^{(k+1)}(x_0, y_0) - a^{(k)}(x_0, y_0) \right\| > \varepsilon_1 \ | \ \left\| b^{(k+1)}(x_0, y_0) - b^{(k)}(x_0, y_0) \right\| > \varepsilon_2$

**do**

**Step 1**: Compute $a^{(k+1)}\ (x_0, y_0)$, $b^{(k+1)}\ (x_0, y_0)$ by using Eq. S (15) with $x_i^j$ and $h_i^{(k)}$;

**Step 2**: Compute $h_i^{(k+1)}$ by using Eq. S (16) with $a^{(k+1)}\ (x_0, y_0)$, $b^{(k+1)}\ (x_0, y_0)$.

**end**

**Supplementary Note 5: 3D positioning compared with Fourier Transform Profilometry (FTP)**

The process of absolute phase image consists of preprocess of raw image, inverse Fourier transform (Supplementary Note 1) and phase unwrapping. Firstly, we design an adaptive filter to smooth the fringe image (Fig. S5 a), resulting a fringe image with a relative less speckles (Fig. S5 b). After inverse Fourier transform, the wrapped phase is extracted as shown in Fig. S5 c. To determine the absolute phase robustly, a global search is proposed including the forward search and backward search. Forward-backward search consists of twice similarity matching operations, in which the forward matching is performed by searching the most similar point in the reference image for a randomly selected point in the measurement image, and the backward search is performed by searching the most similar points in the measurement image for the point determined by the previous forward matching. Therefore, the corresponding point pairs can meet the requirement of global optimum, thus the absolute phase of the whole image can be deduced by subtracting the difference between the phase of point pairs as shown in Fig. S5 d.

The depth estimation is shown in Fig. S5 e by using the process of epipolar constraint and look-up table ($a$, $b$) mentioned in the manuscript. The reconstructed plane is inclined to the reference plane avoiding repeating with the calibrated images, which is used to validate the depth estimation strictly. We removed the titled term to form a plane shown in Fig. S5 f to achieve the comparison with Fourier transform profilometry (FTP).

The proposed positioning algorithm differs from FTP technique in that they have utilized the similarity constraint for subpixel match of corresponding points, since FTP only find the corresponding points with the same phase. Due to the spectrum leakage of phase extraction in Supplementary Note 1, there are significant fluctuations in the FTP results in Fig. S5 g, showing the relatively low spatial resolution. Therefore, our proposed method can achieve a relatively competitive positioning performance compared with the traditional FTP technique, demonstrating the

potential for accurate and robust 3D positioning.

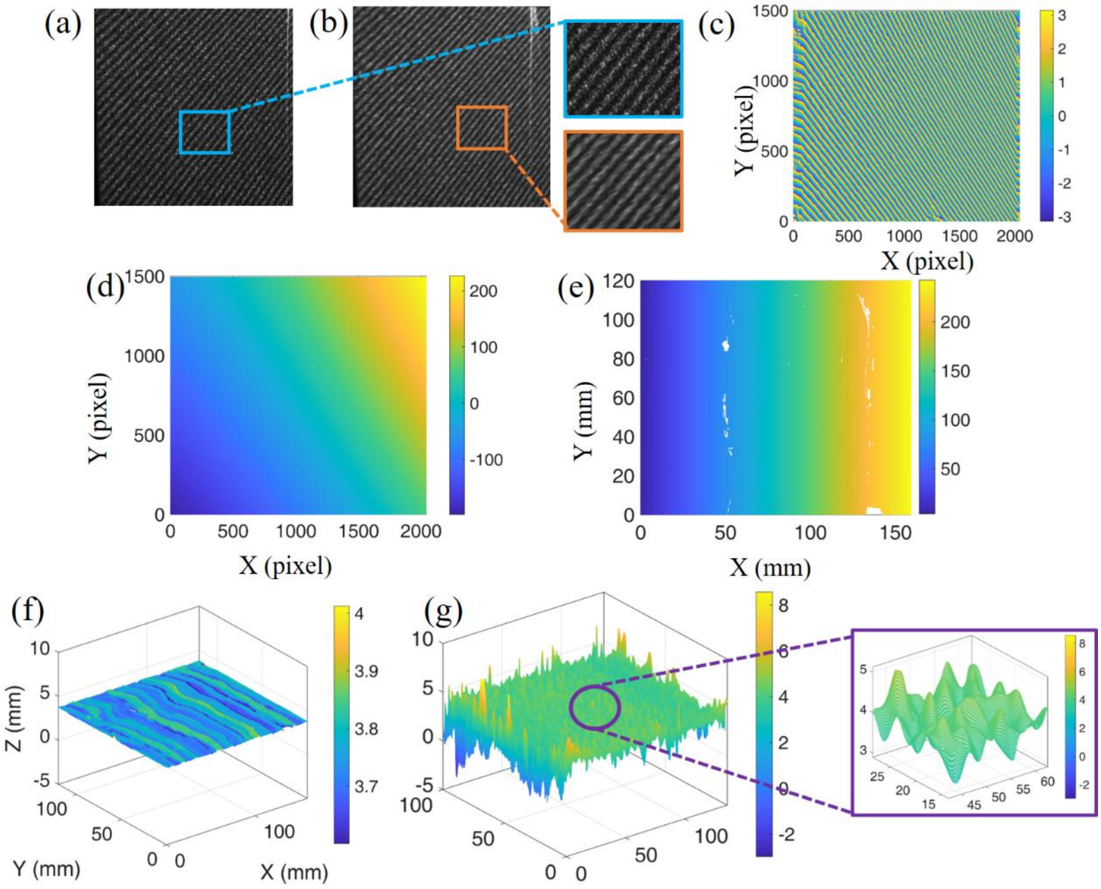


Fig.S5 Comparison with traditional FTP results. (a). Cropped raw image. (b) Image after filtering. The enlarged view shows the comparison with and without filtering. (c) Wrapped phase image. (d) Unwrapped phase image. (e) Reconstructed depth. (f) Remove the titled plane from (e). (g) The reconstructed depth based on FTP. The low resolution data can be seen in the enlarged view.
